# Changes in measures of cognitive function in patients with end-stage kidney disease on dialysis and the effect of dialysis vintage: A longitudinal cohort study

**DOI:** 10.1371/journal.pone.0252237

**Published:** 2021-05-25

**Authors:** Karumathil M. Murali, Judy Mullan, Steven Roodenrys, Hicham I. Cheikh Hassan, Maureen Lonergan

**Affiliations:** 1 Department of Nephrology, Wollongong Hospital, Wollongong, NSW, Australia; 2 School of Medicine, University of Wollongong, Wollongong, NSW, Australia; 3 Centre for Health Research Illawarra Shoalhaven Population (CHRISP), University of Wollongong, Wollongong, NSW, Australia; 4 School of Psychology, University of Wollongong, Wollongong, NSW, Australia; Universita degli Studi Magna Graecia di Catanzaro, ITALY

## Abstract

**Introduction:**

Prevalence of cognitive impairment increases with worsening severity of chronic kidney disease (CKD) and majority of end-stage kidney disease (ESKD) patients on dialysis have cognitive impairment. Trends of cognitive function (CF) in this population are less well known with published studies reporting conflicting results.

**Methods:**

We assessed CF in a cohort of non-dialysis CKD and ESKD patients undergoing dialysis using modified mini-mental state examination (3MS), trail-making test (TMT-A & B) scores and Stroop task, and evaluated demographics, comorbidities and depression using Beck depression inventory at baseline. We repeated tests of CF and depression ≥ 1-year after baseline in both groups and compared change scores in CF and depression between ESKD/ CKD sub-groups. Among ESKD patients we compared change scores between patients with dialysis vintage of <1-year and >1-year. Analysis of covariance was used to adjust for the effect of age on these change scores.

**Results:**

At baseline (N = 211), compared to CKD (N = 108), ESKD (N = 103) patients had significantly worse CF based on 3MS and TMT-A & B scores, and depression scores. On follow-up (N = 160) 3MS scores, especially the memory subscale significantly improved in ESKD, but worsened in CKD, with no significant changes in TMT A /TMT-B, or depression scores after adjusting for age. Among ESKD patients, 3MS, especially memory subscale improved in patients with dialysis vintage <1-year compared to >1-year. The 51 patients who discontinued after baseline assessment had worse baseline CF scores suggesting differential attrition.

**Conclusion:**

Though baseline cognitive scores were worse in ESKD patients on dialysis, compared to CKD, their 3MS, especially memory subscale improved on follow-up. Among ESKD patients, the improvement was significant only in patients who have been on dialysis for less than one-year which may indicate a beneficial effect of clearance of uraemic toxins. Differential attrition of study subjects may have impacted the observed results.

## Introduction

Patients with chronic kidney disease (CKD) have a substantially higher risk of developing cognitive impairment, compared to the general population [1]. This risk steadily increases with worsening renal function [2], and the vast majority of patients with end-stage kidney disease (ESKD) on haemodialysis demonstrate cognitive impairment [1, 3]. Among patients with advanced kidney disease, cerebrovascular disease and accumulation of uraemic toxins are the main contributors [1] to cognitive impairment, which is associated with a higher mortality and morbidity in this population [4].

The increasing prevalence of cognitive impairment among patients with higher relative to lower stages of CKD [2], implies that cognitive decline is likely to worsen over time in this population, which is consistent with the generally progressive course of CKD and vascular disease. However, such progression may be slow and cognitive function was noted to be stable in a CKD cohort over two years [5]. On the other hand, in ESKD patients undergoing dialysis, the intermediate and long-term trajectory of cognitive function may be shaped by the potential benefits of dialytic removal of uraemic toxins versus the potential harm of cerebral ischaemia during haemodialysis treatment [6] and the gradual progression of cerebrovascular disease. Studies evaluating the trends of cognitive performance over time in ESKD patients have yielded conflicting results, with some studies reporting a decline [7–9], while others have not [10–12]. A more recent study evaluating the longitudinal trends of cognitive function domains in prevalent HD patients reported a decline in executive function, but an improvement in memory on follow-up for up to two years [13]. These conflicting results warrants further research to understand the reasons for these diverse findings.

For the current study, we hypothesised that the baseline cognitive function as well as the rate of cognitive decline would be worse in ESKD compared to CKD patients. We also hypothesised that the cognitive decline may be less severe in patients who were relatively new to dialysis, due to the clearance of accumulated uraemic toxins following initiation of dialysis therapy and the higher vascular disease burden in longer-term dialysis patients. The aim of this study, therefore, was to investigate the cognitive function trends in a cohort of CKD and ESKD patients over time and to specifically examine the relationship between the dialysis vintage (time since initiation of dialysis) and changes in cognitive function in the ESKD cohort.

## Materials and methods

### Study design and population

Patients attending renal outpatient clinics for the treatment of CKD and those with ESKD who were undergoing in-centre or home-based dialysis, in an Australian local health district, were invited to participate in the study. Patients were eligible if they were eighteen years or older, had kidney disease and were able to provide informed consent. ESKD patients had to have been on dialysis for at least three months prior to enrolment, to ensure that they were stable from a clinical and biochemical perspective. We did not undertake a formal sample size estimation based on statistical power and anticipated change in test results, because ‘minimal clinically significant difference’ and ‘variance’ of the outcome, which are essential for such calculations [14] were not available due the diverse nature of study designs and results from published studies. Based on local experience with enrolling dialysis patients for clinical studies, we aimed to recruit approximately 50% of the 230 prevalent dialysis patients in the health district. We aimed to recruit approximately equal numbers of patients undergoing dialysis for ESKD and non-dialysis CKD as controls. Patients were excluded if: they had undergone kidney transplantation or were being actively worked up for a living donor transplant; had been diagnosed with dementia or intellectual impairment; had an anticipated life expectancy of less than 12 months as perceived by their primary physician based on their comorbidities, or were on a renal palliative care pathway. To ensure there were no known exclusion criteria or concerns for the patient’s participation in the study, their primary physician was consulted before approaching the patients to request participation and consent. Capacity to consent was informally determined by the researcher and formal questionnaires assessing capacity were not used. Because the test instruments were administered in English, patients who did not speak and read English were also excluded from the study. The study received ethics approval from the authorised ‘University of Wollongong and Illawarra Shoalhaven local health district health and medical human research ethics committee’ (HE14/398–HREC/14/WGONG/90). The consent process as well as the consent forms and test instruments were approved by the ethics committee. When an exclusion criterion was recognized after the informed consent was signed, the patients were excluded from the study.

### Data collection

#### Demographic and clinical data

After receiving informed written consent from the study participants, the research assistants used a combination of patient interviews and reviews of the medical records to collect demographic data (age, gender, race, educational level and income), smoking status and clinical data, including the cause of renal disease, comorbidities (diabetes, hypertension, hyperlipidaemia, ischaemic heart disease, cerebrovascular disease, peripheral vascular disease and lung disease), duration of dialysis in ESKD patients and serum creatinine and estimated glomerular filtration rate (eGFR) at enrolment in the non-dialysis CKD patients.

#### Measures of cognitive function and depression

Cognitive function was assessed using a combination of tests. The modified mini-mental state examination (3MS test) [15] was used to measure the global cognitive scores, and the sub-scales of 3MS test were used to assess the domains of verbal memory and fluency, language and executive function, orientation and visuo-construction and praxis, as outlined by the factor analysis by Rapp et al. [16]. The Trail-making test part A (TMT-A) and part B (TMT-B) were administered to assess the psychomotor speed and executive function [17] and the Stroop colour word interference test was used to evaluate cognitive flexibility and control [18]. Patients were considered to be cognitively impaired if they scored ≤85 in the 3MS test; their TMT scores were 1.5 or more standard deviations above the predicted mean scores adjusted for the patient’s age and educational level; or the Stroop interference score was 1.5 or more standard deviations below the predicted mean scores adjusted for age [3, 19]. Montreal cognitive assessment scale (MoCA) offers a simple and practical tool to detect mild cognitive impairment and dementia by evaluating the domains of Visuo-spatial / executive function, Naming, Memory, Attention, Language, Abstraction, and Orientation [20]. Compared to the Mini-mental state examination (MMSE) instrument, MoCA tool has a high sensitivity (83–90% versus 17–25% with MMSE) but a lower specificity (50–87% versus 100% with MMSE) for the diagnosis of mild cognitive impairment and dementia [20, 21]. The combination of tools we have used assesses all the domains captured by MoCA tool in greater detail. Due to the significant association of depression with cognitive decline in CKD and ESKD patients reported in the literature [22, 23] all of the participants completed the Beck depression inventory (BDI-2). A cut-off score of ≥14 was used to categorise participants as ‘depressed’ [24], and the raw score was used as a covariate in other analyses. We also measured health related quality of life using RAND-36 item short form (SF-36) health survey [25].

Baseline data were collected between January 2015 and June 2016. For ESKD patients receiving haemodialysis, test instruments were administered either before or in the first hour of dialysis to avoid any possible cognitive fluctuations related to dialysis treatment. It has been demonstrated that cognitive performance does not significantly differ between tests done one hour before versus during the first hour of haemodialysis [26]. For non-dialysis CKD patients and home-dialysis patients, who attended the outpatient nephrology clinics, the tests were administered in a single session to minimise the need for multiple visits. The test instruments were administered only if patients were clinically well and not suffering from any acute intercurrent illness.

Follow-up data, comprising of the same measures of cognitive function and depression used at baseline, were collected between one and two years after the baseline data collection. Patients were considered as ‘discontinued’ if their follow-up was interrupted due to death, transplantation, relocation or withdrawal of consent.

### Statistical methods

We used means and standard deviations to describe continuous variables and proportions for discrete variables. We compared baseline demographic and clinical characteristics, as well as the test scores for cognitive function and depression between ESKD and non-dialysis CKD patients using Wilcoxon rank-sum test for continuous variables and Chi-square test for categorical variables. The changes in scores of cognitive function and depression, between baseline and follow-up in the overall study cohort, were compared using the paired signed-ranks test when the change score had a skewed distribution and paired t-test when the score was not significantly skewed. To compare between CKD and ESKD patients, we used Student’s t-test to compare the change scores of markers of cognitive function and depression when the scores were normally distributed and Wilcoxon rank-sum (Mann-Whitney) test when the change scores showed skewed distribution. Due to the significant impact of age on cognitive function, we evaluated the relationship between change scores and the ESKD/CKD status using the analysis of covariance (ANCOVA) model with the participant’s age at enrolment in the study as the covariate and the change score as the dependent variable.

Discontinuation due to death, transplantation, or drop out is an important issue in longitudinal studies of dialysis patients [13] with the risk of biased assessment if the attrition is selective and unbalanced. To understand the patterns of attrition relevant to the study, we compared the baseline scores of cognitive function and depression in participants who discontinued the study with those who completed the follow-up.

To evaluate the effect of dialysis vintage on the cognitive function markers in the ESKD subgroup, we compared the changes in scores between baseline and follow-up, in patients who have been on dialysis for less than 12 months with those who have been on dialysis for a longer duration. We also examined the relationship between change scores (dependent variable) and dialysis vintage categories (<1-year vs >1-year) (independent variable) using ANCOVA models with participant age at enrolment in the study as the covariate. Missing data was handled by list-wise deletion. Statistical significance was set at a two-tailed p-value of <0.05 for all comparisons. The statistical analyses were undertaken using Stata 16.0.

## Results

### Study population

Of the 321 eligible patients (173 CKD and 148 ESKD) invited to participate in the study, 66 (20.6%) declined. Five patients were excluded because they did not fulfil the inclusion criteria, and 28 patients discontinued due to withdrawal of consent, kidney transplantation, or death before baseline data collection. Cognitive function tests were incomplete in eleven patients, leading to a baseline cohort of 211 patients (108 non-dialysis CKD and 103 ESKD, out of which six patients were on home peritoneal dialysis, four on home haemodialysis, and the remaining on in-centre haemodialysis). For non-dialysis CKD patients, the mean creatinine level was 155 ± 99.4umol/L and mean eGFR 45 ± 21.2mL/min. at study enrolment. Among CKD patients, 7.5% had stage 1 CKD, 13.1% had stage 2 CKD, 50.5% had stage 3 CKD, 26.1% had stage 4 CKD, and 2.8% had stage 5 CKD.

**Fig 1:**
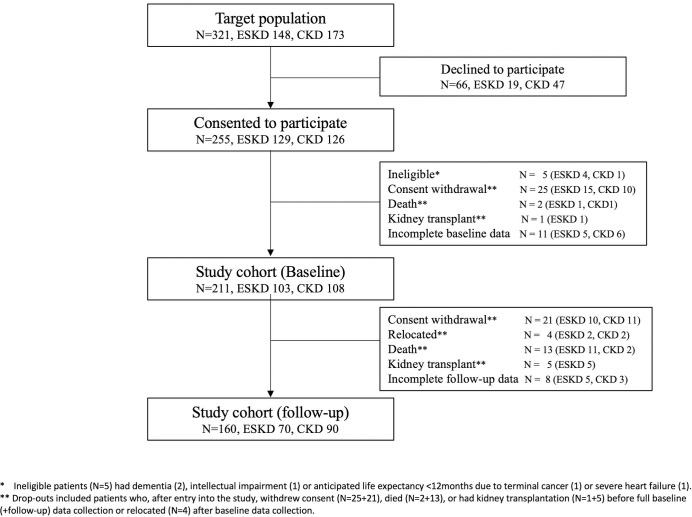
Flow chart of patient participation in the study.

After the baseline data collection was completed, 43 patients (15 CKD, 28 ESKD) discontinued their participation (21 withdrawal of consent, 13 deaths, five transplants and four relocations) and eight patients had incomplete cognitive function tests for the follow-up data collection. This resulted in a follow-up cohort of 160 patients (90 CKD, 70 ESKD). For the ESKD patients who completed follow-up assessments, 21 of the 70 patients (30%) had a dialysis vintage less than one-year (3–12 months), and 49 (70%) patients had been on dialysis for longer than one year at baseline. Follow-up data collection was completed between one and two years after the baseline in all patients and the time intervals in ESKD patients (median 14.9months, IQR 12.7–16.7months, Range 369–677 days) and CKD patients (median 14.5months, IQR 13.6–15.6months, Range 367–689 days) were not significantly different (p = 0.534). The flow chart of patient recruitment and progress through the study is given in Fig 1.

### Baseline demographic and clinical characteristics

The baseline characteristics of the study population and the two sub-groups (ESKD and CKD) are given in Table 1.

**Table 1:** Baseline demographic and clinical characteristics of the study population comparing non-dialysis CKD and ESKD patients.

	Overall population	CKD patients	ESKD patients	p value
	Mean ± SD / %	Mean ± SD / %	Mean ± SD / %	
No of patients	211	108	103	
Age (years)	66.9 ± 11.2	68.1 ± 10.5	65.7 ±11.9	0.058
Gender (M/F)	61.1 / 38.9%	61.1/ 38.9%	61.2 / 38.8%	0.994
Indigenous population (%)	5.3%	2.8%	8.0%	0.093
Education				
<12 years	53.3%	54.6%	52.0%	0.913
12–13 years	8.1%	8.3%	7.8%	
13–15 years	12.4%	13.0%	11.8%	
> = 16 years	26.2%	24.1%	28.4%	
Gross annual income				
<$25,000	58.8%	46.3%	71.8%	**0.003**
$25–50,000	29.4%	40.7%	17.5%	
$50–75,000	6.2%	6.5%	5.8%	
>$75,000	5.7%	6.5%	4.9%	
Smoking status				
Current smoker	9.5%	6.5%	12.6%	0.150
Former smoker	56.4%	62.0%	50.5%	
Non-smoker	34.1%	31.5%	36.9%	
Cause of renal disease				
Diabetic nephropathy	33.9%	30.4%	37.6%	0.067
Hypertension / Vascular disease	20.8%	25.0%	16.5%	
Polycystic kidney disease	4.5%	2.7%	6.4%	
Glomerulonephritis	15.4%	11.6%	19.3%	
Others / Unknown aetiology	25.3%	30.3%	20.2%	
Comorbidity burden				
Diabetes	43.1%	40.6%	45.6%	0.460
Hypertension	90.4%	88.7%	92.2%	0.395
Ischaemic heart disease	37.5%	23.6%	52.0%	**<0.001**
Cerebrovascular disease	15.9%	12.3%	19.6%	0.147
Peripheral vascular disease	21.6%	9.4%	34.3%	**<0.001**
Lung disease	27.9%	25.5%	30.4%	0.429

p values in bold print represent values with <0.05 significance level

The mean age of the study population was 67 years, with the ESKD patients (66 years) tending to slightly younger than the CKD patients (68 years). The majority of the ESKD and CKD (61%) patients were male, and the educational levels were comparable between these subgroups. Annual income was significantly lower in the ESKD compared to CKD patients (p = 0.003). Ischaemic heart disease (p = <0.001) and peripheral vascular disease (p = <0.001) were significantly more prevalent in ESKD patients who also tended to have a higher frequency of diabetic nephropathy (p = 0.067) as the cause of underlying kidney disease. Smoking status and prevalence of comorbidities like diabetes, hypertension, cerebrovascular disease and lung disease were comparable between the CKD and ESKD patient subgroups.

### Baseline measures of cognitive function, depression and health-related quality of life

The baseline scores of cognitive function and depression are provided in Table 2. The scores of the 3MS test, TMT-A and TMT-B, and the difference between TMT-A and B were significantly worse in ESKD compared to CKD patients at baseline, while the Stroop interference scores were not significantly different. Analysis of covariance adjusted for age as a continuous covariate, evaluating the relationship between eGFR at study enrollment and the scores of 3MS test (F (1, 104) = 0.95, p = 0.333), TMT-A (F (1, 104) = 0.11, p = 0.736), and TMT-B (F (1, 103) = 1.23, p = 0.269), showed no statistically significant association. The scores for the 3MS sub-scale [16], pertaining to the verbal memory and fluency, as well as the orientation and visuo-construction were significantly lower in ESKD patients. The proportion of patients with impaired cognitive function, based on the test scores outside the above-described thresholds of 3MS, TMT-A, TMT-B, and Stroop interference scores, was larger in ESKD patients. Depression was significantly higher in ESKD patients compared to CKD patients.

**Table 2:** Baseline markers of cognitive function and depression among the study participants comparing non-dialysis CKD and ESKD patients.

Test instrument used to measure cognitive function / depression	Overall population	CKD patients	ESKD patients	p value*
	(N = 211)	(N = 108)	(N = 103)	
	Mean ± SD / %	Mean + SD / %	Mean + SD / %	
Modified mini-mental state examination (3MS) score	92.1 ± 6.7	93.7 ± 5.4	90.4 ± 7.5	**<0.001**
Cognitive impairment (patients with 3MS score <85%)	14.2%	7.4%	21.4%	**0.004**
3MS—Verbal memory & Fluency domain	25.4 ± 3.4	26.3 ± 2.5	24.6 ± 4.1	**0.004**
3MS—Orientation and visuo-construction domain	19.2 ± 1.8	19.5 ± 0.9	18.8 ± 2.3	**0.030**
3MS—Language-Praxis domain	10.7 ± 0.8	10.7 ± 0.8	10.6 ± 0.9	0.154
3MS—Language and Executive function domain	16.2 ± 2.3	16.4 ± 2.2	16.0 ± 2.3	0.288
Trail making test part A (TMT-A) (sec)	47.8 ± 32.8	37.8 ± 14.3	58.6 ± 42.5	**<0.001**
Cognitive impairment based on age adjusted TMT-A scores	15.4%	4.6%	27.0%	**<0.001**
Trail making test part B (TMT-B) (sec)	122.3 ± 83.1	99.0 ± 58.0	147.3 ± 97.8	**<0.001**
Cognitive impairment based on age adjusted TMT-B scores	18.3%	7.5%	30.0%	**<0.001**
Difference between TMT-A & TMT-B (sec)	74.5 ± 60.2	61.2 ± 49.5	88.6 ± 67.2	**<0.001**
Stroop interference score	-2.2 ± 7.6	-1.4 ± 7.5	- 3.0 ± 7.6	0.076
Cognitive impairment based on age adjusted Stroop scores	13.7%	6.5%	21.2%	**0.002**
Cognitive impairment based on any of the above 3 domains	28.9%	14.0%	43.7%	**<0.001**
Beck depression inventory II score	10.0 ± 8.0	7.9 ± 6.5	12.2 ± 8.7	**<0.001**
Depression (based on BDI II score of 14 or above)	26.4%	13.9%	39.8%	**<0.001**

* p value for difference between CKD and ESKD subgroups estimated by Wilcoxon rank-sum test for continuous variables Chi-square test for difference in proportions.

p values in bold print represent values with <0.05 significance level

Measures of health-related quality of life for the overall sample and the sub-groups are provided in S1 Table. Quality of life domains related to ‘physical functioning’, ‘role limitation due to physical health’, role limitation due to emotional problems’, ‘energy/fatigue’, ‘social functioning’ and ‘general health’ were significantly worse in ESKD patients compared to CKD patients.

### Baseline measures in patients who discontinued follow-up

Table 3 provides a comparison of the baseline scores for the 51 (24.2%) patients (18 CKD, 33 ESKD) who discontinued their participation in the study, with the 160 patients (90 CKD, 70 ESKD), who completed baseline and follow-up data collection. We noted that 3MS and TMT scores were significantly worse in the discontinued patients, compared to those who completed follow-up. Among subgroups, baseline cognitive measures were significantly worse in ESKD patients who discontinued study participation, while among the CKD subgroup, the scores of discontinued patients were worse than patients who completed follow-up, but the difference was not statistically significant. The Stroop interference and depression scores were not significantly different in patients who discontinued the study in the overall population or ESKD/ CKD subgroups.

**Table 3:** Baseline markers of cognitive function and depression among patients who discontinued the study before follow-up assessment compared to patients who completed follow-up assessment, in the overall sample, CKD and ESKD subgroups.

	Overall sample (Total N = 212)			CKD patients. (Total N = 108)			ESKD patients Total N = 104)		
	Discontinued patients	Followed up patients		Discontinued patients	Followed up patients		Discontinued patients	Followed up patients	
	(N = 51)	(N = 160)	p value	(N = 18)	(N = 90)	p value	(N = 33)	(N = 70)	
Modified mini-mental state examination (3MS) score	88.7 ± 8.4	93.2 ± 5.6	**0.001**	91.1 ± 7.5	94.3 ± 4.7	0.119	87.5 ± 8.8	91.8 ± 6.4	**0.018**
Trail making test A (sec)	64.6 ± 55.3	42.4 ± 18.1	**0.005**	41.9 ± 19.2	37.0 ± 13.0	0.470	77.0± 64.2	49.6 ± 21.3	**0.047**
Trail making test B (sec)	167.9 ± 118.3	107.4 ± 61.3	**<0.001**	128.7 ± 80.6	93.0 ± 50.7	0.111	189.3 ± 130.7	126.6 ± 68.9	**0.013**
Difference between TMT-A & TMT-B (sec)	103.3 ± 73.1	65.0 ± 52.2	**<0.001**	86.8 ± 64.5	56.0 ± 44.6	0.064	112.4 ± 76.9	77.0 ± 59.1	**0.010**
Stroop interference score	-3.2 ± 6.8	-1.9 ± 7.8	0.337	-1.5 ± 7.7	-1.4 ± 7.5	0.914	-4.3 ± 6.2	-2.5 ± 8.2	0.353
Beck depression inventory II score	10.3 ± 8.4	9.9 ± 7.8	0.819	6.7 ± 7.1	8.1 ± 6.4	0.197	12.2 ± 8.5	12.2 ± 8.9	0.793

* p value for difference estimated by Wilcoxon rank-sum test for continuous variables

p values in bold print represent values with <0.05 significance level

### Changes in cognitive function and depression scores over time

The changes in scores for the 160 patients who completed both baseline and follow-up assessments are given in Table 4. There was a mild but significant improvement in 3MS scores in the overall study cohort, which was mainly due to the improvement in 3MS scores observed in the ESKD patients and there was a marginal worsening among CKD patients. Further analysis of the 3MS sub-scales [16], showed that there was a significant improvement for only the verbal memory and fluency domain, while the domains of orientation and visuo-construction, praxis and language-executive function showed no significant differences on follow-up, either in the overall cohort or ESKD/ CKD subgroups. The TMT-A, TMT-B, their difference and the Stroop interference score showed no significant difference in the overall study population or in either subgroup. There were also no differences in depression scores over time in the overall cohort and between the subgroups.

**Table 4:** Changes in scores of cognitive function and depression between baseline and follow-up for the overall sample and comparison of change scores between non-dialysis CKD and ESKD subgroups.

	Overall sample (N = 160)		CKD (N = 90)	ESKD (N = 70)	
	Change score	p value*	Change score	Change score	p value**
Modified mini-mental state examination (3MS) score	0.73 ± 4.6	**0.045**	-0.18 ± 3.8	1.9 ± 5.2	**0.004**
3MS—Verbal memory & Fluency domain	0.44 ± 3.2	0.193	-0.02 ± 2.4	1.04 ± 4.0	**0.037**
3MS—Orientation and visuo-construction domain	0.04 ± 1.7	0.548	-0.12 ± 1.4	0.26 ± 2.0	0.438
3MS—Language-Praxis domain	0.08 ± 0.7	0.440	0.0 ± 0.6	0.19 ± 0.8	0.120
3MS—Language and Executive function domain	- 0.08 ± 1.9	0.592	0.04 ± 1.8	- 0.24 ± 2.1	0.348
Trail making test A (sec)	- 1.37 ± 16.7	0.309	- 0.47 + 9.9	- 2.63 + 23.1	0.432
Trail making test B (sec)	- 2.9 + 48.7	0.362	0.18 + 43.1	-7.3 ± 55.9	0.309
Difference between TMT-A & TMT-B (sec)	0.39 ± 46.0	0.899	0.84 ± 42.1	- 2.2 ± 51.4	0.684
Stroop interference score	- 0.41 ± 7.0	0.779	-0.60 ± 7.0	- 0.14 ± 7.1	0.343
Beck depression inventory II score	- 0.16 ± 5.3	0.434	- 0.30 ± 4.6	0.02 ± 6.2	0.577

* p value for change in scores between baseline and follow up for the overall sample estimated by paired sign-rank test for continuous variables with skewed distribution and paired t-test for variables with Normal distribution

** p value for comparison between CKD and ESKD subgroups estimated by Wilcoxon rank-sum test for continuous variables with skewed distribution and two-sample t-test for continuous variables with Normal distribution.

p values in bold print represent values with <0.05 significance level

### Dialysis vintage and changes in cognitive function and depression among ESKD patients

Table 5 provides the results of a comparison of changes in cognitive and depression scores among ESKD patients with a dialysis vintage of <12 months and >12 months at baseline assessment. The improvement in 3MS scores was significantly greater in ESKD patients who had been on dialysis for less than 12 months, compared to those with a longer dialysis vintage. With respect to the 3MS sub-scales, there was significant improvement in the verbal memory and fluency subscale for the overall ESKD dialysis cohort and a comparison of categories, based on dialysis vintage, showed that the improvement was significantly higher in patients who had been on dialysis for less than 12 months. There were no significant changes in any of the other 3MS sub-scales, other cognitive function scores (TMT-A, TMT-B, Stroop interference) or depression scores.

**Table 5:** Changes in scores of cognitive and psychological measures at follow up compared to baseline for the dialysis cohort and comparison of change scores between patients who commenced dialysis within one year compared to patients who have been on dialysis longer than one year.

	Dialysis cohort (N = 70)		On dialysis for < 1 year (N = 21)	On dialysis for > 1 year (N = 49)	
	Change score	p value*	Change score	Change score	p value**
Modified mini-mental state examination (3MS) score	1.9 ± 5.2	**0.003**	4.9 ± 4.9	0.61 ± 4.9	**0.005**
3MS—Verbal memory & Fluency domain	1.04 ± 4.0	**0.031**	2.8 ± 4.5	0.28 ± 3.5	**0.014**
3MS—Orientation and visuo-construction domain	0.26 ± 2.0	0.360	0.86 ± 2.6	0.00 ± 1.7	0.415
3MS—Language-Praxis domain	0.19 ± 0.8	0.120	0.33 ± 1.3	0.12 ± 0.6	0.988
3MS—Language and Executive function domain	- 0.24 ± 2.1	0.326	0.29 ± 2.0	- 0.47 ± 2.1	0.160
Trail making test A (sec)	- 2.6 ± 23.1	0.366	- 3.0 ± 22.5	- 2.4 ± 23.6	0.838
Trail making test B (sec)	- 7.3 ± 55.9	0.309	- 10.4 ± 47.7	- 6.0 ± 59.7	0.775
Difference between TMT-A & TMT-B (sec)	- 2.2 ± 51.4	0.743	- 6.3 ± 40.1	- 0.30 ± 56.1	0.677
Stroop interference score	- 0.14 ± 7.1	0.406	-0.57 ± 10.2	0.07 ± 5.2	0.754
Beck depression inventory II score	0.02 ± 6.2	0.984	- 1.10 ± 4.7	0.55 ± 6.8	0.333

* p value for change in scores between baseline and follow up for the overall dialysis cohort estimated by paired sign-rank test for continuous variables with skewed distribution and paired t-test for variables with Normal distribution

** p value for comparison between dialysis vintage <1year and >1year subgroups estimated by Wilcoxon rank-sum test for continuous variables with skewed distribution and two-sample t-test for continuous variables with Normal distribution.

p values in bold print represent values with <0.05 significance level

### Analyses of covariance adjusting for age

**Fig 2:**
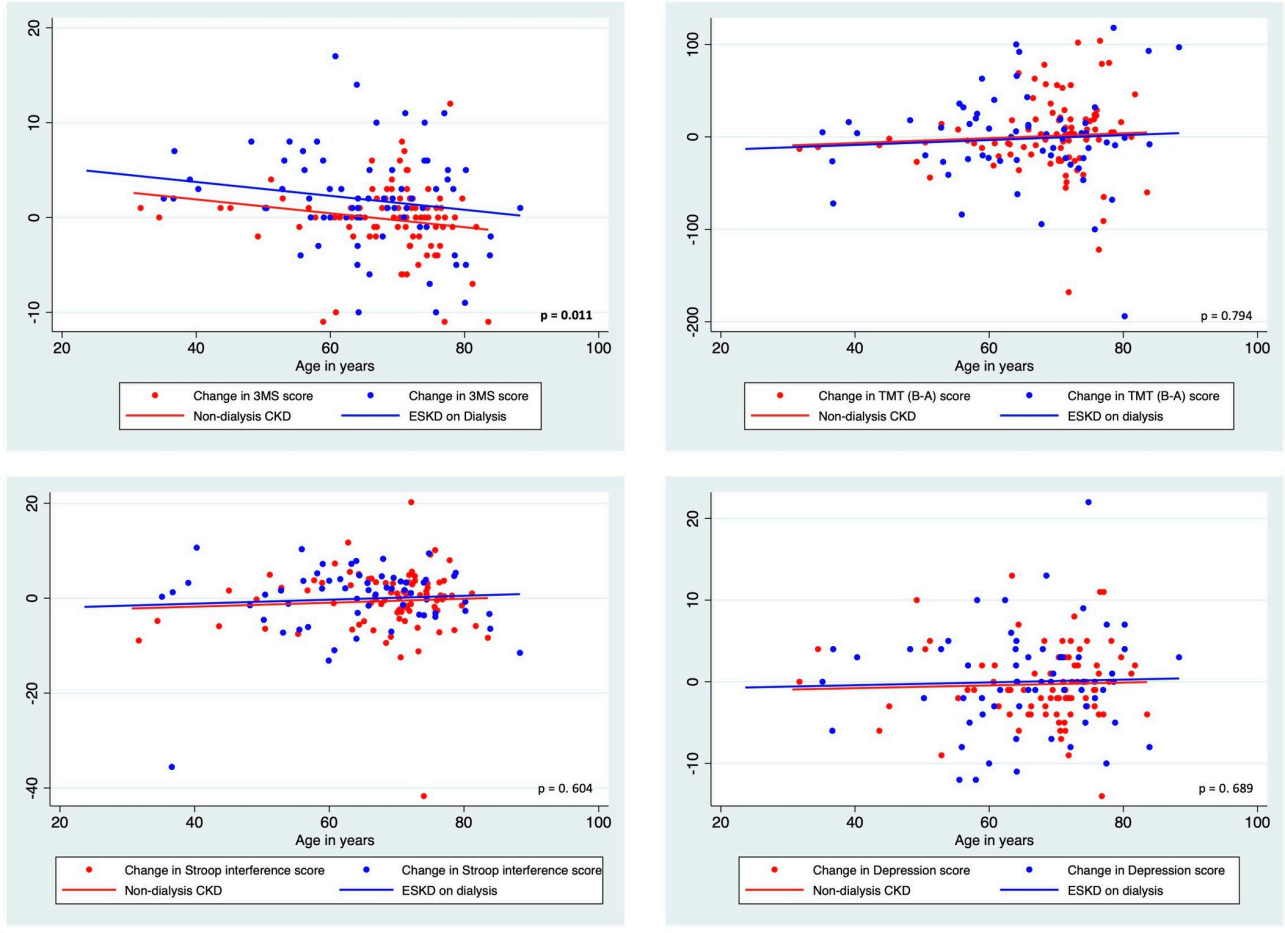
Change scores of cognitive function and depression in CKD and ESKD patients with fitted regression lines from ANCOVA models using age as a continuous covariate.

**Fig 3:**
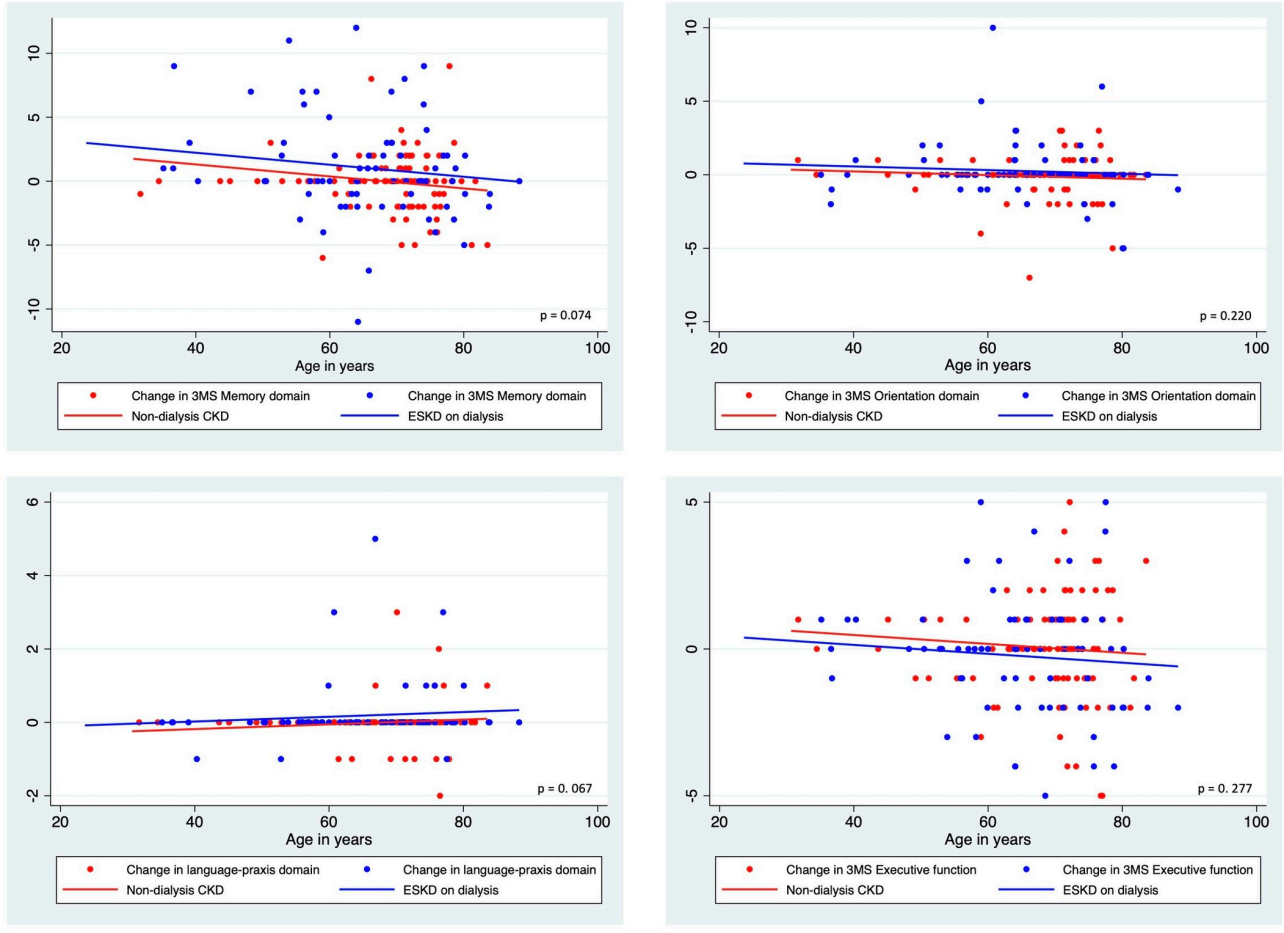
Change scores of memory, orientation, praxis and executive function domains of cognitive function in CKD and ESKD patients with fitted regression lines from ANCOVA models using age as a continuous covariate.

To evaluate whether the participant age might have impacted upon the observed changes in cognitive markers, we examined ANCOVA models with the change in cognitive function scores as the dependent variable, ESKD/ CKD status as the independent variable, and age as a continuous covariate. Our evaluation found that the change in 3MS aggregate scores was significantly different between CKD and ESKD patients (F (1, 157) = 6.65, p = 0.011), which was mainly due to the change in the 3MS memory sub-scale scores which approached significance (F (1, 157) = 3.24, p = 0.074). The remaining 3MS sub-scales and markers of other cognitive function tests and depression were not significantly different between CKD and ESKD patients. Graphs showing the distribution of change scores, with the fitted regression lines from the ANCOVA and the corresponding p values, are shown in Figs 2 and 3.

**Fig 4:**
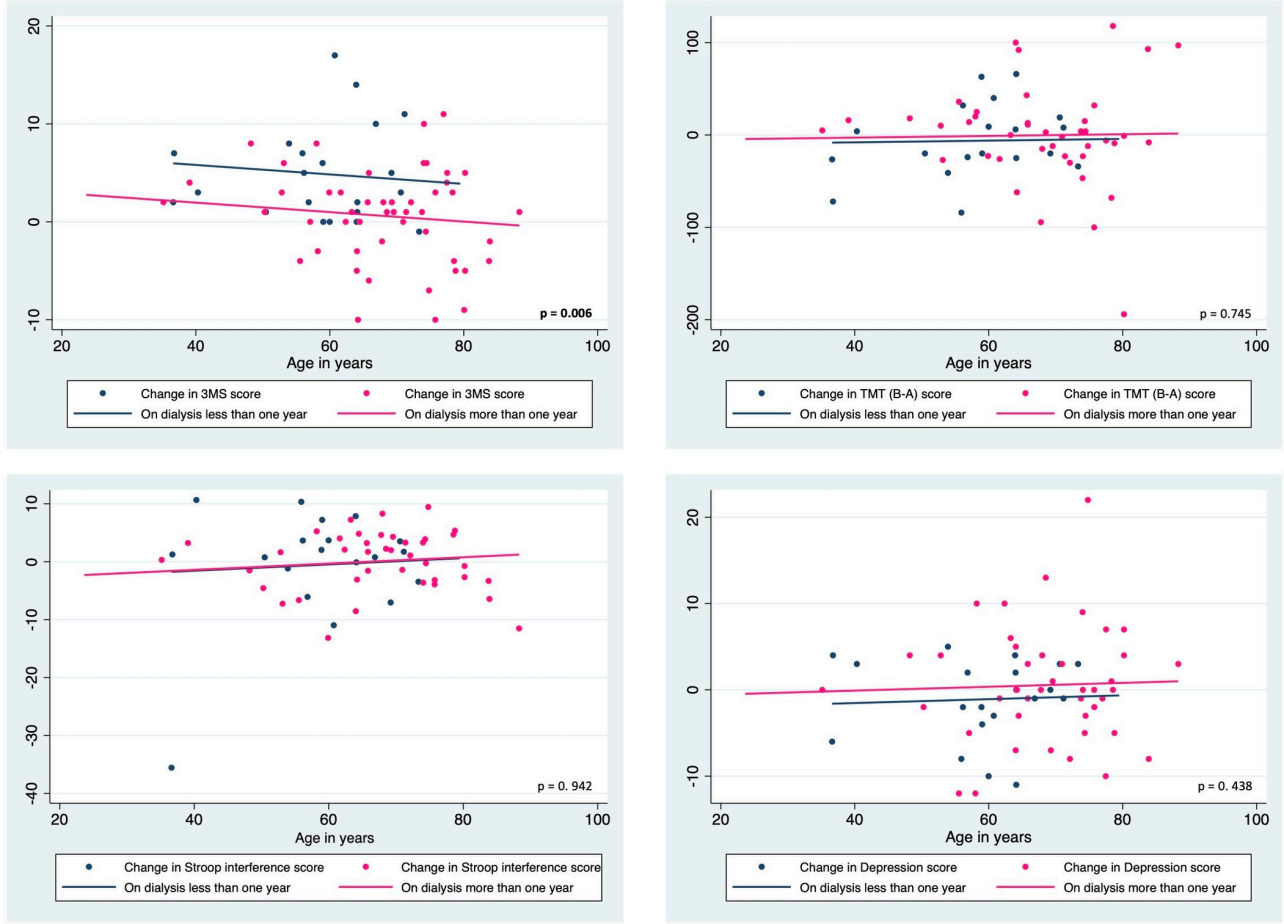
Change scores of cognitive function and depression in ESKD patients with dialysis vintage <1-year and >1-year with fitted regression lines from ANCOVA models using age as a continuous covariate.

**Fig 5:**
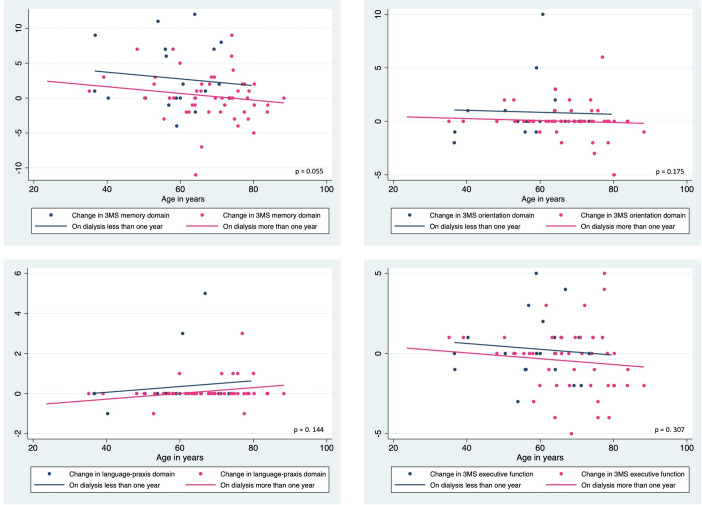
Change scores of memory, orientation, praxis and executive function domains of cognitive function in ESKD patients with dialysis vintage <1-year and >1-year with fitted regression lines from ANCOVA models using age as a continuous covariate.

A similar analysis evaluating the change scores between categories of dialysis vintage (<1-year versus >1-year) showed that change in 3MS aggregate scores was significantly different between the categories (F (1, 67) = 7.95, p = 0.006), which was mainly due to the change in the 3MS memory sub-scale scores which approached significance (F (1, 67) = 3.82, p = 0.055). The remaining 3MS sub-scales and measures of other cognitive function tests and depression were not significantly different between dialysis vintage categories. Graphs showing the distribution of change scores with the fitted regression lines from the ANCOVA and the corresponding p values are shown in Figs 4 and 5.

## Discussion

Our results showed that cognitive function scores were significantly worse in ESKD compared to non-dialysis CKD patients at baseline, which is consistent with published literature [1–3]. However, upon follow-up, the ESKD patients receiving dialysis, and in particular those who had been receiving dialysis for less than 12 months had mild but significant improvement in their overall 3MS scores, which appeared to be related to an improvement in the verbal memory and fluency sub-scale scores. Measures of executive function and cognitive flexibility, as well as depression, were also significantly worse in ESKD compared to CKD patients at baseline. However, there were no significant differences on follow-up compared to baseline. We also noted selective attrition of participants, with a higher proportion of patients with worse baseline cognitive function discontinuing their study participation, especially among ESKD patients. To interpret our findings, we will discuss the conflicting results from the published studies regarding the cognitive trends in this population and how the characteristics of our study population might explain our findings.

Direct comparisons of studies evaluating the cognitive function trends in ESKD and CKD patients are difficult to interpret due to the diverse nature of test instruments used to measure cognitive function, the variable duration of follow-up, the inconsistent reporting of patient dropouts and variations in study design. A study using the Mini-Mental State Examination (MMSE) score to assess cognitive function with one year of follow-up [7] and another using the Montreal Cognitive Assessment (MOCA) tool with two years of follow-up [9] reported a worsening trend of cognitive function in ESKD patients undergoing haemodialysis. On the other hand, another study, which used a panel of cognitive measures (3MS, TMT-A and TMT-B scores) to evaluate cognitive trends in haemodialysis patients with a focus on patient frailty did not observe any deterioration in cognitive performance on follow-up in the overall cohort, though frail patients showed a greater cognitive decline compared to non-frail patients [10]. We used a similar panel of cognitive test instruments, and observed no deterioration in cognitive function on follow-up, contrary to our hypothesis about the cognitive deterioration in ESKD population. A randomised trial comparing two phosphate binders over a two-year period reported a declining trend of cognitive function using cognitive drug research (CDR) tools in both intervention and control arms [8]. However, the measures of cognitive function in this study were biased towards speed of processing, which the above studies have not assessed. The differences in the characteristics, sensitivity and specificity of cognitive function instruments may partly account for the variations in the cognitive trends reported in the literature and observed in our study. In addition, our results may have been influenced by the characteristics of our study cohort, who had higher baseline cognitive scores and a lower prevalence of cognitive impairment compared to many published studies in this population [2, 7–10, 13].

Our findings of an improvement in the aggregate 3MS scores and the verbal memory and fluency sub-scale in dialysis patients were unexpected and contrary to our hypotheses that the rate of cognitive decline would be worse in ESKD compared to CKD patients. However, our findings are partly consistent with the published literature. For instance, a study which compared cognitive changes over time between patients receiving nocturnal dialysis and kidney transplant, reported a modest but significant improvement in auditory verbal learning, but no improvements in any of the other cognitive function tests among the dialysis cohort [12]. Similarly, another study, evaluating 314 dialysis patients, found a decline in their executive function and yet a modest improvement in memory over two years of follow-up [13]. In the latter study, the authors suggested that the learning effect due to the repeated administration of the same cognitive tests and confounding due to survival bias, resulting from the drop-out of patients at higher risk of memory decline, may explain the unexpected improvement in memory. To account for the selective attrition, the authors undertook competing risk analysis for death, transplantation and other censoring events using joint models, but there was little overall difference between the results of the linear mixed models and joint models [13].

Dialysis patients with cognitive impairment have increased mortality [4] resulting in higher drop-out due to death. However, non-mortality drop-outs are also known to be higher in cognitively impaired individuals [27], and any hypothesis in longitudinal studies can be affected by selective attrition and survival bias [28]. In our study, baseline cognitive measures were worse in patients who dropped-out, compared to patients completing follow-up, especially among ESKD patients. The patients who dropped-out may have had a greater risk of experiencing cognitive decline if they had remained in the study. It should be noted however, that attrition of up to one-third of the participants is not uncommon in longitudinal studies which have evaluated cognitive function in patients with kidney disease [8, 10, 13]. Differences in attrition may partly explain the disparity between our findings and the published literature, and it is important to note that some studies, reporting declining cognitive trends, have not reported the patient drop-out rates or characteristics [7, 9].

Improvement in 3MS scores in the ESKD sub-group in our study was mostly driven by improvement in the verbal memory and fluency sub-scale scores in patients with a dialysis vintage <12 months. It is conceivable that the physiological benefits of an improved biochemical profile may be most evident in the first year of dialysis, due to the transition from a severely uraemic to less uraemic milieu interieur after initiation of dialysis. Vascular disease burden and cognitive function were significantly worse in the ESKD group in our study suggesting a role for vascular disease in the cognitive decline in the study population. However, it has been suggested that accumulation of uraemic toxins may be more important in the pathogenesis of cognitive impairment in kidney disease patients than pro-atherosclerotic factors, since interventions targeting cardiovascular risk factors have little or no effect on CKD-associated cognitive decline [29]. The improvement in memory we observed may therefore be due to a physiological benefit resulting from the removal of uraemic neurotoxins with dialysis. Even though severe uraemic symptoms improve soon after commencing dialysis, stabilisation of nutritional status and uraemic neuropathy can take up to one year after the initiation of dialysis [30]. However, Drew et al., identified no relationship between dialysis vintage and decline of memory or executive function [13]. The disparate finding in our study may be due to variations in the characteristics of our sample, differential attrition patterns or analysis models, and we can be more confident about our finding if future studies reproduce similar results. We did not observe any significant difference in the non-memory sub-scale scores of 3MS test or other cognitive instrument scores between ESKD patients versus CKD patients or between dialysis vintage categories among ESKD patients.

Our study has several limitations. Our study cohort comprised of only two-thirds of potentially eligible patients, and it is possible that patients with better cognitive function may have been preferentially recruited. It is well known that patients with a higher disease burden and greater risk of adverse outcomes are over-represented in the non-participant group leading to selection bias in prospective studies [28]. However, the control population of CKD patients drawn from the same socio-demographic and geographical setting used in our study provided an opportunity for direct comparison between CKD and ESKD patients to test our hypothesis. We should nevertheless appreciate that only a minority of CKD patients progress to dialysis requiring ESKD and there are inherent imbalances in direct comparison of the CKD and ESKD patients. This imbalance is apparent from the differences in baseline characteristics between groups and we have undertaken multivariate analysis to account for the relevant variables. It should also be stressed that the observed improvements in cognitive parameters in dialysis patients were small and of uncertain clinical significance. However, it is reasonable to interpret within the limitations of the study, that there is no deterioration in cognitive function in dialysis population over the period of observation. The sensitivity and specificity of instruments used to measure cognitive function would impact upon the reliability of our reported findings. A more robust battery of tests administered by a psychologist may have enabled us to classify the cognitive domains with greater accuracy but several studies in kidney disease patients have used the panel of cognitive measures we have used because it is simpler and more practical to administer in a routine clinical setting. When neuropsychological assessments are repeated in the same patient, learning effect can modify the follow-up results in some participants [31], which should be considered in the interpretation of our results. However, such learning effects would be expected to be similar in the two patient groups and learning effects cannot explain the difference in changes in cognitive function between CKD and ESKD groups. The relatively high differential drop-out rate of patients with worse cognitive function after baseline data collection may have introduced survival bias by selecting patients with a lower risk of cognitive decline, but our drop-out rate was, in fact, lower than what is reported in other longitudinal studies in this population. The drop-out, however, doesn’t invalidate the observation of improved memory domain scores in patients who have a dialysis vintage less than one year compared to those on dialysis for a longer duration.

In conclusion, we observed that baseline cognitive function scores pertaining to memory and executive function were worse in ESKD patients, compared to CKD patients. However, we found that global cognitive scores and scores pertaining to verbal memory and fluency improved on follow-up for ESKD patients, compared to CKD patients, which was largely driven by the improvement in patients with a dialysis vintage of less than one year. These findings may indicate that the memory domain may be responsive to biochemical changes while other cognitive domains including executive function, may be largely dictated by cerebrovascular disease. Our findings call for more research in this area focusing on the early effect of dialysis on cognitive function domains.

## Supporting information

S1 Checklist STROBE statement—checklist of items that should be included in reports of *cohort studies*. (DOCX)

S1 Table Baseline scores of health-related quality of life among the study participants comparing non-dialysis CKD and ESKD patients. (DOCX)

## References

[1] Drew DA, Weiner DE, Sarnak MJ. Cognitive Impairment in CKD: Pathophysiology, Management, and Prevention. Am J Kidney Dis. 2019;74(6):782–90. Epub 2019/08/06. 10.1053/j.ajkd.2019.05.017 31378643 PMC7038648

[2] Kurella M, Chertow GM, Fried LF, Cummings SR, Harris T, Simonsick E, et al. Chronic kidney disease and cognitive impairment in the elderly: the health, aging, and body composition study. J Am Soc Nephrol. 2005;16(7):2127–33. Epub 2005/05/13. 10.1681/ASN.2005010005 . 15888561

[3] Murray AM, Tupper DE, Knopman DS, Gilbertson DT, Pederson SL, Li S, et al. Cognitive impairment in hemodialysis patients is common. Neurology. 2006;67(2):216–23. Epub 2006/07/26. 10.1212/01.wnl.0000225182.15532.40 . 16864811

[4] Griva K, Stygall J, Hankins M, Davenport A, Harrison M, Newman SP. Cognitive impairment and 7-year mortality in dialysis patients. Am J Kidney Dis. 2010;56(4):693–703. Epub 2010/08/31. 10.1053/j.ajkd.2010.07.003 . 20800327

[5] Gronewold J, Todica O, Seidel UK, Volsek M, Kribben A, Bruck H, et al. Cognitive Performance Is Highly Stable over a 2-Year-Follow-Up in Chronic Kidney Disease Patients in a Dedicated Medical Environment. PLoS One. 2016;11(11):e0166530. Epub 2016/11/12. 10.1371/journal.pone.0166530 27835681 PMC5106023

[6] Findlay MD, Dawson J, Dickie DA, Forbes KP, McGlynn D, Quinn T, et al. Investigating the Relationship between Cerebral Blood Flow and Cognitive Function in Hemodialysis Patients. J Am Soc Nephrol. 2019;30(1):147–58. Epub 2018/12/12. 10.1681/ASN.2018050462 30530658 PMC6317612

[7] Bossola M, Antocicco M, Di Stasio E, Ciciarelli C, Luciani G, Tazza L, et al. Mini Mental State Examination over time in chronic hemodialysis patients. J Psychosom Res. 2011;71(1):50–4. Epub 2011/06/15. 10.1016/j.jpsychores.2011.01.001 . 21665013

[8] Altmann P, Barnett ME, Finn WF. Cognitive function in Stage 5 chronic kidney disease patients on hemodialysis: no adverse effects of lanthanum carbonate compared with standard phosphate-binder therapy. Kidney Int. 2007;71(3):252–9. Epub 2006/10/13. 10.1038/sj.ki.5001932 . 17035945

[9] Iyasere O, Okai D, Brown E. Cognitive function and advanced kidney disease: longitudinal trends and impact on decision-making. Clin Kidney J. 2017;10(1):89–94. Epub 2017/06/24. 10.1093/ckj/sfw128 28638609 PMC5469575

[10] McAdams-DeMarco MA, Tan J, Salter ML, Gross A, Meoni LA, Jaar BG, et al. Frailty and Cognitive Function in Incident Hemodialysis Patients. Clin J Am Soc Nephrol. 2015;10(12):2181–9. Epub 2015/11/18. 10.2215/CJN.01960215 26573615 PMC4670760

[11] Sharma A, Yabes J, Al Mawed S, Wu C, Stilley C, Unruh M, et al. Impact of Cognitive Function Change on Mortality in Renal Transplant and End-Stage Renal Disease Patients. Am J Nephrol. 2016;44(6):462–72. Epub 2016/11/01. 10.1159/000451059 27798939 PMC5143182

[12] Dixon BS, VanBuren JM, Rodrigue JR, Lockridge RS, Lindsay R, Chan C, et al. Cognitive changes associated with switching to frequent nocturnal hemodialysis or renal transplantation. BMC Nephrol. 2016;17:12. Epub 2016/01/24. 10.1186/s12882-016-0223-9 26801094 PMC4722762

[13] Drew DA, Weiner DE, Tighiouart H, Duncan S, Gupta A, Scott T, et al. Cognitive Decline and Its Risk Factors in Prevalent Hemodialysis Patients. Am J Kidney Dis. 2017;69(6):780–7. Epub 2017/01/31. 10.1053/j.ajkd.2016.11.015 28131531 PMC5441943

[14] Hickey GL, Grant SW, Dunning J, Siepe M. Statistical primer: sample size and power calculations-why, when and how? Eur J Cardiothorac Surg. 2018;54(1):4–9. Epub 2018/05/15. 10.1093/ejcts/ezy169 29757369 PMC6005113

[15] Teng EL, Chui HC. The Modified Mini-Mental State (3MS) examination. The Journal of clinical psychiatry. 1987;48(8):314–8. Epub 1987/08/01. . 3611032

[16] Rapp SR, Espeland MA, Hogan P, Jones BN, Dugan E. Baseline experience with Modified Mini Mental State Exam: The Women’s Health Initiative Memory Study (WHIMS). Aging Ment Health. 2003;7(3):217–23. Epub 2003/05/31. 10.1080/1360786031000101201 . 12775404

[17] Gordon NG. The Trail Making Test in neuropsychological diagnosis. Journal of clinical psychology. 1972;28(2):167–9. Epub 1972/04/01. 10.1002/1097-4679(197204)28:2&lt;167::aid-jclp2270280212&gt;3.0.co;2-x . 5019979

[18] Graf P, Uttl B, Tuokko H. Color- and picture-word Stroop tests: performance changes in old age. Journal of clinical and experimental neuropsychology. 1995;17(3):390–415. Epub 1995/05/01. 10.1080/01688639508405132 . 7650102

[19] Metanalysis tables for the trail making test (TMT) and the Stroop test (Golden version, interference condition). In: Mitrushina. KBB M., Razani J., D’Elia L.F., editor. Handbook of normative data for neuropsychological assessment. 2nd ed. New Yorkl: Oxford University Press; 2005. p. 648–83.

[20] Nasreddine ZS, Phillips NA, Bédirian V, Charbonneau S, Whitehead V, Collin I, et al. The Montreal Cognitive Assessment, MoCA: a brief screening tool for mild cognitive impairment. J Am Geriatr Soc. 2005;53(4):695–9. Epub 2005/04/09. 10.1111/j.1532-5415.2005.53221.x . 15817019

[21] Smith T, Gildeh N, Holmes C. The Montreal Cognitive Assessment: validity and utility in a memory clinic setting. Can J Psychiatry. 2007;52(5):329–32. Epub 2007/06/05. 10.1177/070674370705200508 . 17542384

[22] Seidel UK, Gronewold J, Volsek M, Todica O, Kribben A, Bruck H, et al. The prevalence, severity, and association with HbA1c and fibrinogen of cognitive impairment in chronic kidney disease. Kidney Int. 2014;85(3):693–702. Epub 2013/10/04. 10.1038/ki.2013.366 . 24088956

[23] Agganis BT, Weiner DE, Giang LM, Scott T, Tighiouart H, Griffith JL, et al. Depression and cognitive function in maintenance hemodialysis patients. Am J Kidney Dis. 2010;56(4):704–12. Epub 2010/08/03. 10.1053/j.ajkd.2010.04.018 20673602 PMC2943330

[24] Steer RA, Ball R, Ranieri WF, Beck AT. Dimensions of the Beck Depression Inventory-II in clinically depressed outpatients. Journal of clinical psychology. 1999;55(1):117–28. Epub 1999/04/01. 10.1002/(sici)1097-4679(199901)55:1&lt;117::aid-jclp12&gt;3.0.co;2-a . 10100838

[25] Hays RD, Morales LS. The RAND-36 measure of health-related quality of life. Ann Med. 2001;33(5):350–7. Epub 2001/08/09. 10.3109/07853890109002089 . 11491194

[26] Drew DA, Tighiouart H, Scott TM, Lou KV, Shaffi K, Weiner DE, et al. Cognitive performance before and during hemodialysis: a randomized cross-over trial. Nephron Clin Pract. 2013;124(3–4):151–8. Epub 2013/12/18. 10.1159/000356393 24335582 PMC4440830

[27] Matthews FE, Chatfield M, Freeman C, McCracken C, Brayne C. Attrition and bias in the MRC cognitive function and ageing study: an epidemiological investigation. BMC Public Health. 2004;4:12. Epub 2004/04/29. 10.1186/1471-2458-4-12 15113437 PMC419705

[28] Tripepi G, Jager KJ, Dekker FW, Zoccali C. Selection bias and information bias in clinical research. Nephron Clin Pract. 2010;115(2):c94–9. Epub 2010/04/22. 10.1159/000312871 . 20407272

[29] Viggiano D, Wagner CA, Martino G, Nedergaard M, Zoccali C, Unwin R, et al. Mechanisms of cognitive dysfunction in CKD. Nat Rev Nephrol. 2020;16(8):452–69. Epub 2020/04/03. 10.1038/s41581-020-0266-9 . 32235904

[30] Cabrera VJ, Hansson J, Kliger AS, Finkelstein FO. Symptom Management of the Patient with CKD: The Role of Dialysis. Clin J Am Soc Nephrol. 2017;12(4):687–93. Epub 2017/02/06. 10.2215/CJN.01650216 28148557 PMC5383375

[31] McCaffrey RJ, Ortega A, Haase RF. Effects of repeated neuropsychological assessments. Arch Clin Neuropsychol. 1993;8(6):519–24. Epub 1993/11/01. . 14591991

