# Supplementary material for: Changes in measures of cognitive function in patients with end-stage kidney disease on dialysis and the effect of dialysis vintage: A longitudinal cohort study
Source: PLoS One. 2021 May 25;16(5):e0252237. doi: 10.1371/journal.pone.0252237 (PMC8148363; doi:10.1371/journal.pone.0252237)
Supplement: S1 Checklist — (DOCX) [file pone.0252237.s001.docx]

STROBE Statement—Checklist of items that should be included in reports of ***cohort studies***

Changes in measures of cognitive function in patients with end stage kidney disease on dialysis and the effect of dialysis vintage: A longitudinal cohort study with chronic kidney disease patients as controls

|  | Item No | Recommendation |
| --- | --- | --- |
| **Title and abstract** | 1 | (*a*) Indicate the study’s design with a commonly used term in the title or the abstract  Page 1 |
|  |  | (*b*) Provide in the abstract an informative and balanced summary of what was done and what was found  Pages 3-4 |
| Introduction | | |
| Background/rationale | 2 | Explain the scientific background and rationale for the investigation being reported  Pages 5-6 |
| Objectives | 3 | State specific objectives, including any prespecified hypotheses  Page 6 |
| Methods | | |
| Study design | 4 | Present key elements of study design early in the paper  Pages 6-7 |
| Setting | 5 | Describe the setting, locations, and relevant dates, including periods of recruitment, exposure, follow-up, and data collection  Pages 6-8 |
| Participants | 6 | (*a*) Give the eligibility criteria, and the sources and methods of selection of participants. Describe methods of follow-up  Pages 6,8 |
|  |  | (*b*) For matched studies, give matching criteria and number of exposed and unexposed  *Not applicable* |
| Variables | 7 | Clearly define all outcomes, exposures, predictors, potential confounders, and effect modifiers. Give diagnostic criteria, if applicable  Pages 7-8 |
| Data sources/ measurement | 8* | For each variable of interest, give sources of data and details of methods of assessment (measurement). Describe comparability of assessment methods if there is more than one group  Pages 7-8 |
| Bias | 9 | Describe any efforts to address potential sources of bias  Page 9 |
| Study size | 10 | Explain how the study size was arrived at  Page 6, 9 |
| Quantitative variables | 11 | Explain how quantitative variables were handled in the analyses. If applicable, describe which groupings were chosen and why  Pages 8,9 |
| Statistical methods | 12 | (*a*) Describe all statistical methods, including those used to control for confounding  Pages 8,9 |
|  |  | (*b*) Describe any methods used to examine subgroups and interactions  Pages 8,9 |
|  |  | (*c*) Explain how missing data were addressed  Page 9 |
|  |  | (*d*) If applicable, explain how loss to follow-up was addressed  Page 9 |
|  |  | (*e*) Describe any sensitivity analyses  Not applicable |
| Results | | |
| Participants | 13* | (a) Report numbers of individuals at each stage of study—eg numbers potentially eligible, examined for eligibility, confirmed eligible, included in the study, completing follow-up, and analysed  Pages 9,10, Figure 1 |
|  |  | (b) Give reasons for non-participation at each stage  Figure 1 |
|  |  | (c) Consider use of a flow diagram  Figure 1 |
| Descriptive data | 14* | (a) Give characteristics of study participants (eg demographic, clinical, social) and information on exposures and potential confounders  Page 10, Table 1, Table 2, |
|  |  | (b) Indicate number of participants with missing data for each variable of interest  Information on discontinued patients with no follow-up data in page 14, Table 3 |
|  |  | (c) Summarise follow-up time (eg, average and total amount)  Page 10 |
| Outcome data | 15* | Report numbers of outcome events or summary measures over time  Pages 16, Table 4, Table 5 |
| Main results | 16 | (*a*) Give unadjusted estimates and, if applicable, confounder-adjusted estimates and their precision (eg, 95% confidence interval). Make clear which confounders were adjusted for and why they were included  Pages 16, 18, Table 4, Table 5 |
|  |  | (*b*) Report category boundaries when continuous variables were categorized  Not applicable |
|  |  | (*c*) If relevant, consider translating estimates of relative risk into absolute risk for a meaningful time period  Not applicable |
| Other analyses | 17 | Report other analyses done—eg analyses of subgroups and interactions, and sensitivity analyses  Pages 16, 18, Table 4, Table 5, Page 20, Figure 2, Figure 3 |
| Discussion | | |
| Key results | 18 | Summarise key results with reference to study objectives  Page 21 |
| Limitations | 19 | Discuss limitations of the study, taking into account sources of potential bias or imprecision. Discuss both direction and magnitude of any potential bias  Pages 24, 25 |
| Interpretation | 20 | Give a cautious overall interpretation of results considering objectives, limitations, multiplicity of analyses, results from similar studies, and other relevant evidence  Pages 23-25 |
| Generalisability | 21 | Discuss the generalisability (external validity) of the study results  Page 24, 25 |
| Other information | | |
| Funding | 22 | Give the source of funding and the role of the funders for the present study and, if applicable, for the original study on which the present article is based  Given in section of declarations after references |

*Give information separately for exposed and unexposed groups.

**Note:** An Explanation and Elaboration article discusses each checklist item and gives methodological background and published examples of transparent reporting. The STROBE checklist is best used in conjunction with this article (freely available on the Web sites of PLoS Medicine at http://www.plosmedicine.org/, Annals of Internal Medicine at http://www.annals.org/, and Epidemiology at http://www.epidem.com/). Information on the STROBE Initiative is available at http://www.strobe-statement.org.
