# Supplementary material for: Changes in measures of cognitive function in patients with end-stage kidney disease on dialysis and the effect of dialysis vintage: A longitudinal cohort study
Source: PLoS One. 2021 May 25;16(5):e0252237. doi: 10.1371/journal.pone.0252237 (PMC8148363; doi:10.1371/journal.pone.0252237)
Supplement: S1 Table — (DOCX) [file pone.0252237.s002.docx]

|  | S1 Table. Baseline scores of health-related quality of life among the study participants comparing non-dialysis CKD and ESKD patients | | | | |
| --- | --- | --- | --- | --- | --- |
|  |  |  |  |  |  |
|  |  |  |  |  |  |
|  | Domain of health-related quality of life | Overall population | CKD patients | ESKD patients | p value* |
|  |  | (N=211) | (N=108) | (N=103) |  |
|  |  | Mean + SD / % | Mean + SD / % | Mean + SD / % |  |
|  |  |  |  |  |  |
|  | QOL 1: Physical functioning | 53.1 + 28.6 | 62.8 + 25.7 | 42.8 + 27.3 | **<0.001** |
|  | QOL 2: Role limitation due to physical health | 41.4 + 40.4 | 56.1 + 39.2 | 25.8 + 35.6 | **<0.001** |
|  | QOL 3: Role limitation due to emotional problems | 66.5 + 41.8 | 77.1 + 37.1 | 55.2 + 43.7 | **<0.001** |
|  | QOL 4: Energy / Fatigue | 45.2 + 19.2 | 49.4 + 17.7 | 40.5 + 19.7 | **<0.001** |
|  | QOL 5: Emotional well-being | 43.5 + 14.0 | 45.0 + 14.6 | 42.0 + 13.3 | 0.093 |
|  | QOL 6: Social functioning | 74.9 + 25.6 | 82.5 + 22.4 | 66.9 + 26.5 | **<0.001** |
|  | QOL 7: Pain | 69.3 + 24.4 | 70.3 + 24.5 | 68.2 + 24.4 | 0.537 |
|  | QOL 8: General health | 44.4 + 22.3 | 53.0 + 20.5 | 35.2 + 20.5 | **<0.001** |
|  |  |  |  |  |  |
|  | * p value for difference between CKD and ESKD subgroups estimated by Wilcoxon rank-sum test for continuous variables Chi-square test for difference in proportions.  p values in bold print represent values with <0.05 significance level | | | | |
